# Optimization of tumor spheroid model in mesothelioma and lung cancers and anti-cancer drug testing in H2052/484 spheroids

**DOI:** 10.18632/oncotarget.28134

**Published:** 2021-11-23

**Authors:** Dylan A.J. Gendre, Edis Ameti, Wolfram Karenovics, Nadja Perriraz-Mayer, Frédéric Triponez, Véronique Serre-Beinier

**Affiliations:** ^1^Division of Thoracic and Endocrine Surgery, University Hospitals and University of Geneva, 1211 Geneva 4, Switzerland

**Keywords:** cancer patient, lung tumor, pleura mesothelioma, tumor spheroid, chemosensitivity

## Abstract

Advanced lung cancers and mesothelioma remain incurable diseases. Despite some promising new therapy strategies, predicting whether an individual patient will be sensitive to a given therapy is challenging. The purpose of this study is to establish and evaluate the efficiency of a three-dimensional spheroid model of human thoracic cancer in predicting the efficacy of drugs.

Human mesothelioma and lung tumor spheroids were established from cell lines and primary cells derived from the patient. The growth kinetics and cell viability of microtumors were assessed using spheroid size and intracellular ATP level. The sensitivity of the mesothelioma spheroids to the cisplatin or cisplatin/pemetrexed combination was determined.

We determined that studying the kinetics of the spheroid growth for 15 days after seeding 1000 cells/well in a 96-well plate was optimal. Monitoring the growth kinetic and intracellular ATP of spheroids allowed the identification of early changes in spheroid viability. Finally, we validated this model by measuring a dose-dependent reduction in the cell viability of mesothelioma H2052/484 spheroids treated with both first-line treatments, cisplatin and the cisplatin/pemetrexed combination. In conclusion, we have developed a three-dimensional spheroid model of thoracic tumor cells useful for tailoring the medical treatment to the specific characteristics of each patient.

## INTRODUCTION

Cancer is the second leading cause of death globally, accounting for an estimated 9.9 million deaths in 2020. Lung cancers and mesothelioma represent 18.3% of all these cancer-related deaths. Advanced lung cancer and mesothelioma remain incurable diseases.

Malignant mesothelioma tumors are heterogeneous tumors with a complex pattern of molecular changes, including genetic, chromosomic, and epigenetic alterations. Of all the malignant mesothelioma types, the malignant pleural mesothelioma (MPM) is the most common form. It is a particularly aggressive asbestos-related disease with a one-year median survival. The therapeutic options for this pathology are extremely limited, with the first-line regimen, a combination of cisplatin (or carboplatin) and pemetrexed, only increasing survival by about 3 months [1]. Recently, the addition of vascular endothelial growth factor (VEGF) antibodies (bevacizumab) to this basic treatment improved overall survival in patients (MAPS study) [2]. Furthermore, in the past few years, several studies have addressed the possibility of using immunotherapies to treat patients with mesothelioma. Based on results from several clinical trials [3–6], the National Comprehensive Cancer Network (NCCN) Clinical Practice Guidelines in Oncology (NCCN guidelines) recommended nivolumab with or without ipilimumab (both immune checkpoint inhibitors) as preferred treatment option (category 2A) in first-line for patients with biphasic or sarcomatoid histology and in second-line or later MPM settings.

Lung cancer is the leading cause of cancer deaths and the second most commonly diagnosed cancer [7]. Lung cancers are classified into two main types: non-small-cell lung carcinoma (NSCLC), which accounts for 80–85 percent of all lung cancers, and small-cell lung carcinoma (SCLC), which accounts for 10–15 percent. The latter is a more aggressive and rapidly-progressing subtype with an often poor prognosis. NSCLC can be further classified into three sub-types: adenocarcinomas, squamous-cell carcinomas, and large-cell carcinomas. The most common histological subtype of lung cancer is adenocarcinoma, which accounts for 30% of all lung cancers. Current lung cancer treatments include surgical resection, chemotherapy, and radiation therapy, all of which have severe side effects and provide only marginal overall survival benefits. The introduction of anti-angiogenic therapies in combination with chemotherapy, has resulted in a marginally improved median overall survival of advanced NSCLC to 12 months [8]. The identification of distinct molecular subsets amenable to targeted therapies, as well as the early success of immune checkpoint inhibitors, has been one of the most significant therapeutic advances [9–12].

During the past decade, cancer therapy has moved into an era of precision medicine and personalized therapy. Various combination treatments are being researched, and some of them have entered clinical practice, with some promising preliminary results [13, 14]. Despite this, it is challenging to predict whether an individual patient will be sensitive to a given therapy, what mechanism is likely to underlie possible resistance, and what alternative treatment could overcome resistance.

There is a need for good clinical models that make it possible to assess treatment efficacy for an individual patient. The ideal model system maintains the heterogeneity and complexity of the original tumor, has an intact tumor microenvironment, and is expandable at a reasonable cost (in time and money). *In vitro* three-dimensional (3-D) tumour models, including patient-derived tumor organoid (PDO) or spheroid models, have been developed successfully from different human cancers (colon, prostate, pancreatic, bladder and breast cancers [15–18]), including lung carcinoma [19, 20]. They have been proven to accurately replicate the diversity of human cancer biology and to closely recapitulate tissue architecture and function [19–24]. They are more relevant than two-dimensional (2-D) monolayer cell cultures for the screening of anti-cancer treatments [24, 25] and they could be useful for assessing the development of acquired therapeutic resistance [26]. They are cost-effective, relatively easy to develop, and they can be cultured for long periods, so are advantageous for the evaluation of anti-cancer agents. Standardizing the methodologies for using 3D cell culture in medium- to high-throughput screens would be invaluable [27].

The purpose of this study is to evaluate the reproducibility and accuracy of a multicellular tumor spheroid model of lung cancer and mesothelioma to predict the clinical efficacy of drugs. We chose this model because the tumor-derived spheroids are more amenable to high-throughput drug screening than organoids for two main reasons:

- they are easier to spread into large cultures with reproducibility in shapes and sizes.
- the extracellular matrix found in spheroids is produced by their own cells. The organoid formation, on the other hand, requires the use of exogenous extracellular matrices (such as matrigel) secreted primarily by mouse sarcoma cells. This matrice’s lot-to-lot variability and non-human-derived nature limit the use of organoids as humanized platforms.

Over the past 4 decades, different spherical cancer models were described without clear definition of each of them. In 2015, Weiswald LB et al., proposed a rational classification of the four most commonly used spherical cancer models: the multicellular tumor spheroid model (MCTS); the tumorospheres; the tissue-derived tumor spheres and the organotypic multicellular spheroids [28].

Using commercially available cell lines and primary patient-derived cells, we created malignant pleural mesothelioma (MPM) and lung tumor spheroids *in vitro* and validated these models to test the therapeutic efficiency of both first-line treatment. According to the classification of Weisswald [28], the tumor spheroids established in this study were MCTS, obtained by culture of cancer cell lines in nonadherent conditions. To simplify the reading of the article, they were called tumor spheroids.

This model could be used in clinical practice to optimize drug screening for each patient. It could also be used in the research laboratory to discover new therapeutic targets and develop novel drugs.

## RESULTS

### Development of the spheroid culture method on MPM and lung adenocarcinoma cells

First, we studied the formation of multicellular tumor spheroids in 96-well round-bottom plates coated with agarose using tumor cell lines and primary cells derived from human MPM (H2052, H2052/484, and H2452) and from human lung adenocarcinoma (LuCa1, LuCa61, LuCa62) in order to determine the best seeding and culture conditions. These spheroids were generated from single-cell suspension culture in conventional fetal bovine serum (FBS)–supplemented RPMI medium without a supply of an exogenous extracellular matrix (ECM). The scaffold-free technique was preferred because it facilitates the cell aggregation, the continuous deposit of the ECM proteins produced by the cells, and the spheroid generation [29].

**Figure 1:**
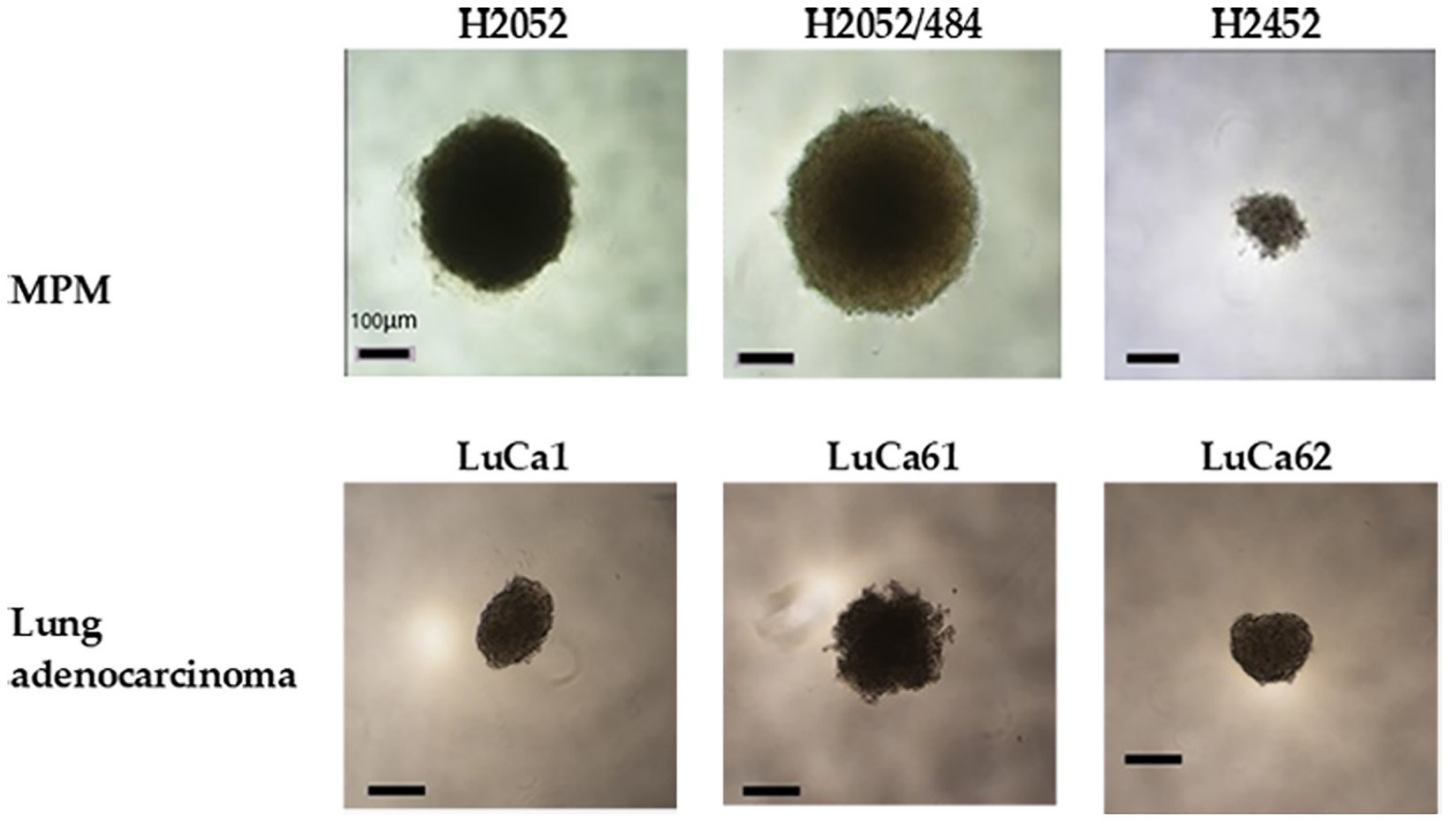
Multicellular spheroids were generated from MPM and lung adenocarcinoma cell populations. Spheroids generated with 1000 cells per spheroid and cultured for 13 days in complete RPMI medium at 37°C and 5% CO_2_ showed different shapes and sizes. Representative images of H2052, H2052/484, H2452, LuCa1, LuCa61, and LuCa62 spheroids viewed from above by inverted phase contrast microscopy. Scale bar represents 100 μm.

As shown in Figure 1, a vast difference in the ability of cells to compact into tight spheroids was observed after 13 days of culture of spheroid obtained after seeding 1000 cells/well. In order to facilitate reading, the term “1000 cells/spheroid” will be used to represent spheroids obtained after seeding 1000 cells/well. The H2452 and LuCa61 cells did not compact but remained in a state of loose aggregation. The H2452 spheroids were small (diameter < 100 μm) and clear. LuCa1 and LuCa62 cells generated compact spheroids, but of irregular shape. The spheroids obtained with the 3 lung adenocarcinoma primary cells (LuCa1, LuCa61 and LuCa62) had a diameter of approximately 100 μm. The MPM H2052 and H2052/484 cells generated tight spheroids with regular shape and diameters between 200 and 300 μm.

**Figure 2:**
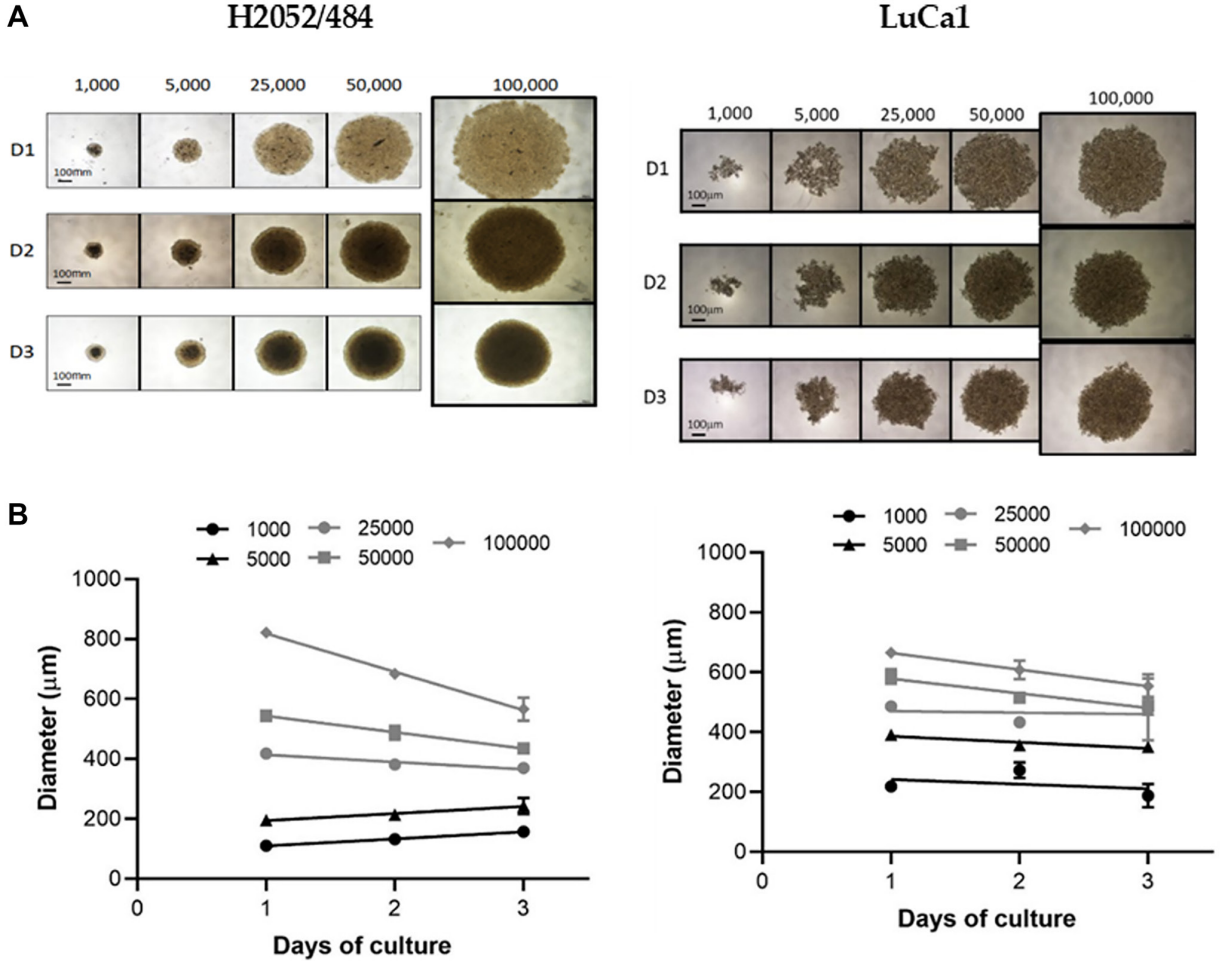
Spheroid morphology and size for increasing seeding densities. (**A**) Representative images and (**B**) spheroid diameters of the MPM H2052/484 (left) and the lung adenocarcinoma LuCa1 (right) spheroids grown at increasing seeding densities (from 10^3^ to 10^5^ cells/spheroid) between days 1 and 3. Scale bar represents 100 μm.

Then, to determine the minimal number of seeding cells required to assess growth and viability, we tested H2052/484 and LuCa1 for spheroid-forming capacity of 1000, 5000, 25000, 50000, and 100000 cells per spheroid. These two cell populations were representative of human MPM and lung adenocarcinoma cell populations, generating high and small spheroids, respectively. Figure 2A shows representative phase contrast micrographs obtained at days 1, 2, and 3 after seeding. At day 1, H2052/484 cells had aggregated; LuCa1 cells remained in a state of loose aggregation. By day 3, H2052/484 cells had formed spheroids with a tight, ideal shape (Figure 2A, left) with clear boundaries. LuCa1 spheroids (Figure 2A, right) were less compact than H2052/484 spheroids and had irregular shapes. The diameters of the spheroids increased with the number of cells seeded (Figure 2B). By day 3, the diameter of H2052/484 spheroids ranged from 157 ± 8 μm to 566 ± 38 μm for 1000 and 100000 cells respectively. The diameter of LuCa1 spheroids ranged from 188 ± 40 μm to 554 ± 40 μm for 1000 and 100000 cells respectively. H2052/484 and LuCa1 spheroids became tighter and more compact after 3 days of culture. For H2052/484 spheroids, the diameter decreased from 418 ± 2 μm by day 1 to 369 ± 11 μm by day 3 for 25000 seeding cells (decrease of 12%), and from 822 ± 12 μm by day 1 to 566 ± 38 μm by day 3 for 100000 seeding cells (decrease of 31%). The diameter of LuCa1 spheroids decreased from 586 ± 24 μm by day 1 to 488 ± 30 μm by day 3 for 50000 seeding cells (decrease of 17%), and from 665 ± 16 μm by day 1 to 554 ± 40 μm by day 3 for 100000 seeding cells (decrease of 17%).

Seeding 1000 cells/well was sufficient to form a spheroid with a diameter of 100 μm at day 3.

**Figure 3:**
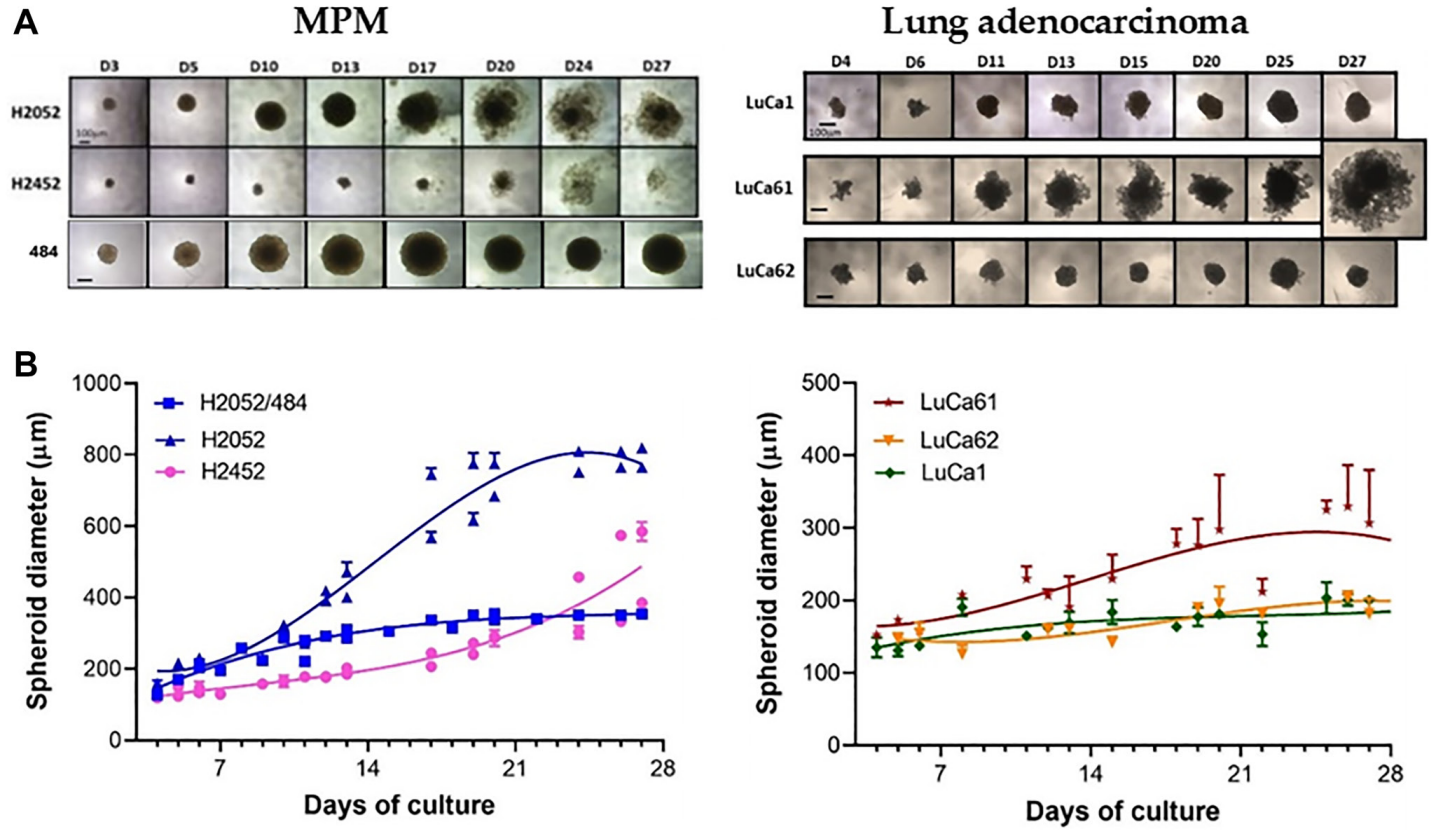
MPM and lung adenocarcinoma spheroid growth kinetic. (**A**) Representative images of the MPM (left) and the lung adenocarcinoma (right) spheroids grown at the optimized seeding densities of 1000 cells/well between days 1 and 27. The scale bar represents 100 μm. (**B**) Growth kinetics represented in spheroid diameters over 24 days at the optimal seeding densities (1000 cells/spheroid). The data represent the mean ± SD of 2 independent experiments for each cell density.

We analyzed the kinetics of spheroid growth of the 3 MPM cell lines (H2052/484, H2452, H2052) and the 3 lung adenocarcinoma (LuCa1, LuCa61, LuCa62) primary cells for 27 days, seeding 1000 cells/spheroid (Figure 3). The size of the spheroids increased over time from day 3 of inoculation for all the cells studied. The growth of the spheroids continued until 20–25 days of culture for H2052/484, LuCa1, and LuCa62 spheroids. At day 17, some cells were observed to have disaggregated from the outer layer of the H2052 spheroids. The same disaggregation phenomenon was observed for H2452 spheroids from day 17. LuCa61 cells aggregated into loose clusters, growing from day 3 to day 11. At day 13, disaggregated cells were identified. The disaggregation process increased over time from day 13 to day 27. The H2052/484 cells formed more compact spheroids, growing for 3 weeks after seeding, with no disaggregation process identified by day 27. Our data showed that the growth curve of spheroids was different for each cell line and primary cells. Comparing the growth curves of the cells studied, except the LuCa61 cells, we observed an increase in the size of spheroid without disaggregation, indicative of a proliferation phase, from day 3 to day 15.

### Intracellular ATP (ATPi) measurement

**Figure 4:**
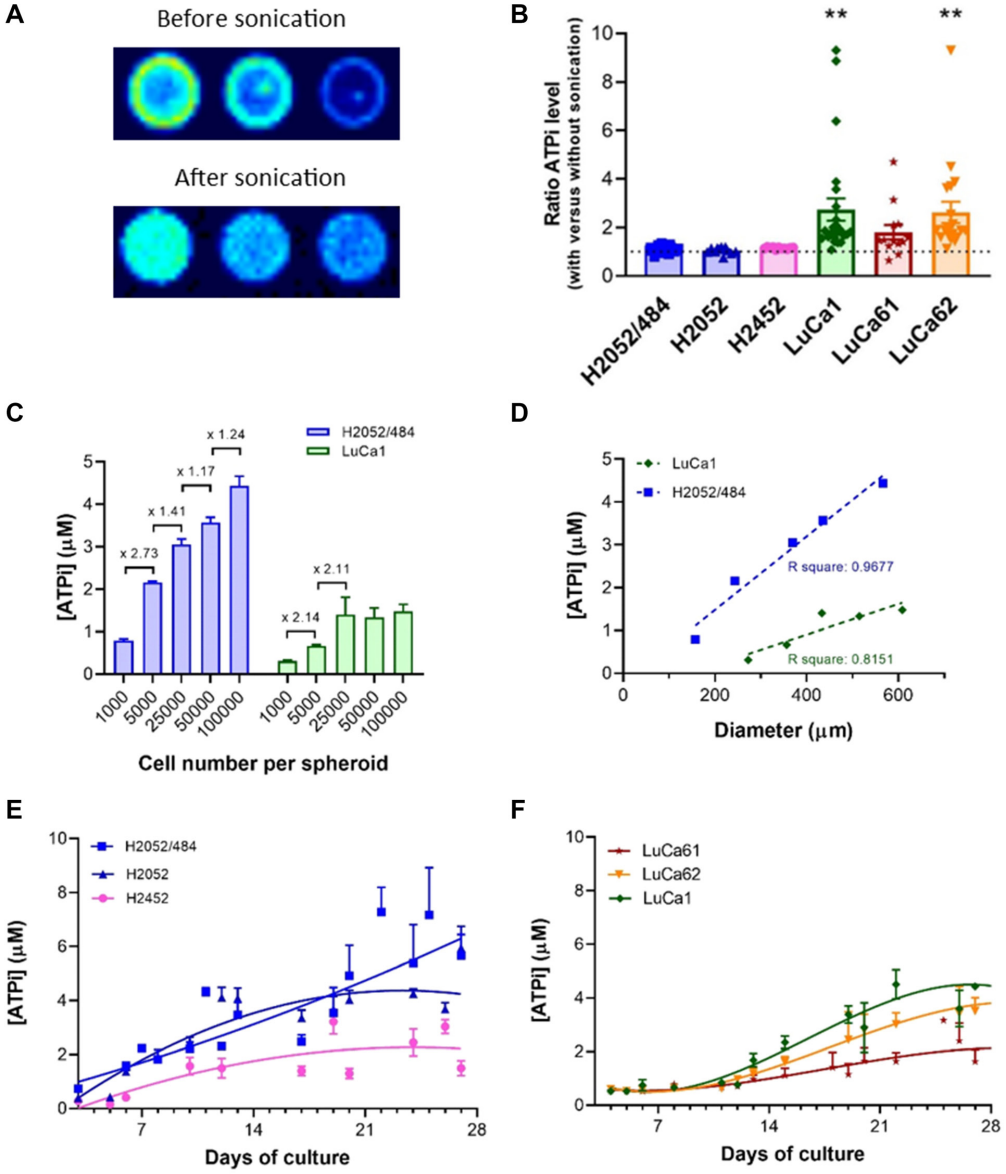
Cell viability measurement using an ATP-based luminescence assay. (**A**) Representative images of the luminescent signals of 3 H2052/484 spheroids grown at the seeding densities of 750 cells/spheroid at day 3 before and after sonication. (**B**) Ratio of ATPi levels measured before and after sonication of H2052/484, H2052, H2452, LuCa1, LuCa61, and LuCa62 spheroids after seeding 750 cells/spheroid. The data represent the mean ± SEM of 3 independent experiments. The ATPi levels measured after sonication were increased compared with those before sonication and statistically significant for LuCa1 and LuCa62 spheroids (^**^ *P* < 0.01). (**C**) ATPi levels of H2052/484 and LuCa1 spheroids at day 3 post-seeding of 10^3^ – 10^5^ cells/spheroid. The ATPi levels increased with cell number per spheroid and were significantly different between 10^3^ cells/spheroid and 10^5^ cells/spheroid (^*^ *P* < 0.05). The data represent the mean ± SD of 2 independent experiments for each cell density. (**D**) Correlation of the ATPi levels and the spheroid size of H2052/484 and LuCa1 spheroids at day 3 post-seeding of 750 cells/spheroid. Kinetics of ATPi of MPM (**E**) and lung adenocarcinoma (**F**) spheroids over 27 days at the optimal seeding densities (1000 cells/spheroid). The data represent the mean ± SD of 3 independent experiments.

ATP is a major metabolite of living cells rapidly lost in dead cells. Cellular ATP levels can provide reliable estimates of the cell viability, including cell survival and cell growth. We assessed the ATPi content of spheroids using a luminescent ATP detection assay kit. The method described by the manufacturer was adapted to optimize it. First, ATP content was measured from MPM spheroids 3 days after seeding 750 cells/well. As shown in Figure 4A for H2052/484 spheroids, the signal of luminescence was not homogeneous in the well.

A halo was detected on the periphery. This signal may have been due to the reflection of the luminescent signal on the white border or to the fluorescence signal of the white plate. A luminescent point was also detected in the center of the well, corresponding to the part of the spheroid that could not be disaggregated during the lysis step. We also noted that agarose in the wells decreased the sensitivity of ATP measurement. We optimized the method by adding a step of sonication, to increase the dissociation efficiency of the lysis step, and by measuring the luminescence signal after transfer of spheroids into an uncoated black 96-well plate. After optimization, we obtained a homogeneous luminescence signal as shown in Figure 4A (bottom), a sign that the spheroid had been properly dissociated. As shown in Figure 4B, for each cell population, the level of ATPi after sonication was found to be higher than that without sonication, indicating a higher lysis rate of the cells. For the MPM spheroids, the estimated level of ATPi after sonication was slightly higher than without the sonication step: 1.12 ± 0.02, 1.05 ± 0.04, and 1.15 ± 0.01 for H2052/484, H2052 and H2452 respectively. For the adenocarcinoma primary cells, the level of ATPi estimated after sonication was around 2-fold higher than without sonication: 2.74 ± 0.46 (*p* = 0.0040), 1.81 ± 0.30, and 2.62 ± 0.43 (*p* = 0.0056) for LuCa1, LuCa61, and LuCa62 respectively. To determine the optimal size of the spheroid required to assess the level of ATPi, we studied the ATPi levels of spheroids formed from increasing number of cells. To simplify the reading of the results, only the data obtained with the H2052/484 and Luca1 cell populations representative of the MPM cell lines and of the primary cells from lung adenocarcinoma are presented. As observed in Figure 4C and 4D, after 3 days of culture the ATPi levels for both cell populations were linearly related to the number of viable cells and the diameter of spheroid. A luminescent signal can be measured with spheroids formed from 1000 cells with a diameter between 157 and 273 μm. We observed a saturation of the luminescent signal with spheroids formed from 25000 cells and more (Figure 4C). As shown in Figure 4E and 4F, ATPi levels increased with time in human cancer cell cultures correlating with growth kinetics (Figure 3B). The ATPi levels of the spheroids increased over time from day 3 of inoculation until 20–25 days of culture for all the cell populations studied. The ATPi levels of LuCa61 spheroids were smaller than the other lung adenocarcinoma cells whereas their spheroid diameters were the highest. Images of the LuCa61 spheroids (Figure 3A) suggested that the increase in the diameter of the spheroids may be related to the detachment of cells from the outer layer and not to the growth of the spheroids. The increase of the ATPi levels of LuCa61 spheroids over time showed that the LuCa61 cells proliferate similarly to the other lung adenocarcinoma cells.

### Study of the effect of standard chemotherapeutic treatments

To evaluate whether our spheroid models could assess the effect of anticancer therapy, we determined the effectiveness of both first-line treatments, cisplatin and the cisplatin/pemetrexed combination, on H2052/484 spheroids. Three days after seeding, H2052/484 spheroids were treated for 3 hours with cisplatin (50, 100, or 200 μM; as described in the literature [30–32]) or with the cisplatin/pemetrexed combination (50/200 μM, 100/400 μM or 200/800 μM) and cultured for an additional 17 days after washes.

**Figure 5:**
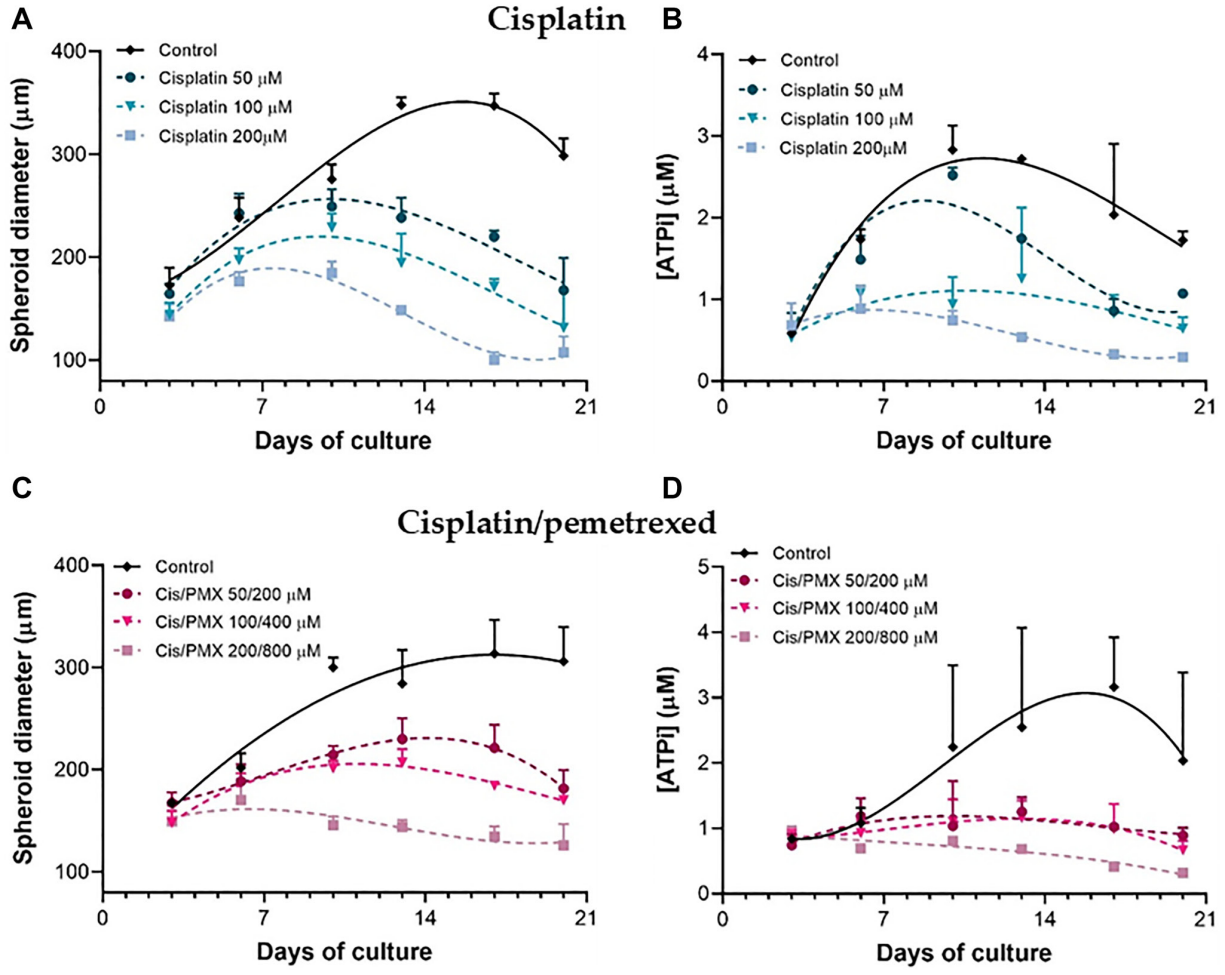
Dose-dependent effect of cisplatin and cisplatin/pemetrexed treatments on H2052/484 spheroids. At day 3 post-seeding, H2052/484 spheroids (1000 cells/spheroid) were treated for 3 hours with different concentrations of cisplatin (50, 100, or 200 μM) (**A**, **B**) or cisplatin/pemetrexed combination (50/200, 100/400, or 200/800 μM) (**C**, **D**). After washes, the spheroids were cultured for 17 days, the growth (A, C) and the ATPi kinetics (B, D) were assessed. The data represent the mean ± SD of 3 independent experiments. ^*^ *P* < 0.05; ^**^ *P* < 0.01 comparing treated groups with control group.

The growth (Figure 5A and 5C) and the ATPi level (Figure 5B and 5D) of H2052/484 spheroids incubated with both treatments were dose-dependently reduced compared with those of non-treated spheroids starting at 50 μM of cisplatin and 50 μM/200 μM of the cisplatin/pemetrexed combination. 17 days after the treatment, we observed a huge shrinkage or a complete disintegration of the H2052/484 spheroids (Supplementary Figure 1) for the highest concentrations of cisplatin and cisplatin/pemetrexed combination. The cisplatin/pemetrexed combination seems to affect the growth and the viability of the H2052/484 spheroids more efficiently than the cisplatin treatment alone. No increases in the size or ATPi level of the spheroids treated with the 200 μM cisplatin/800 μM pemetrexed concentrations were observed, suggesting that these concentrations completely blocked the growth and the viability of the treated spheroids.

**Figure 6:**
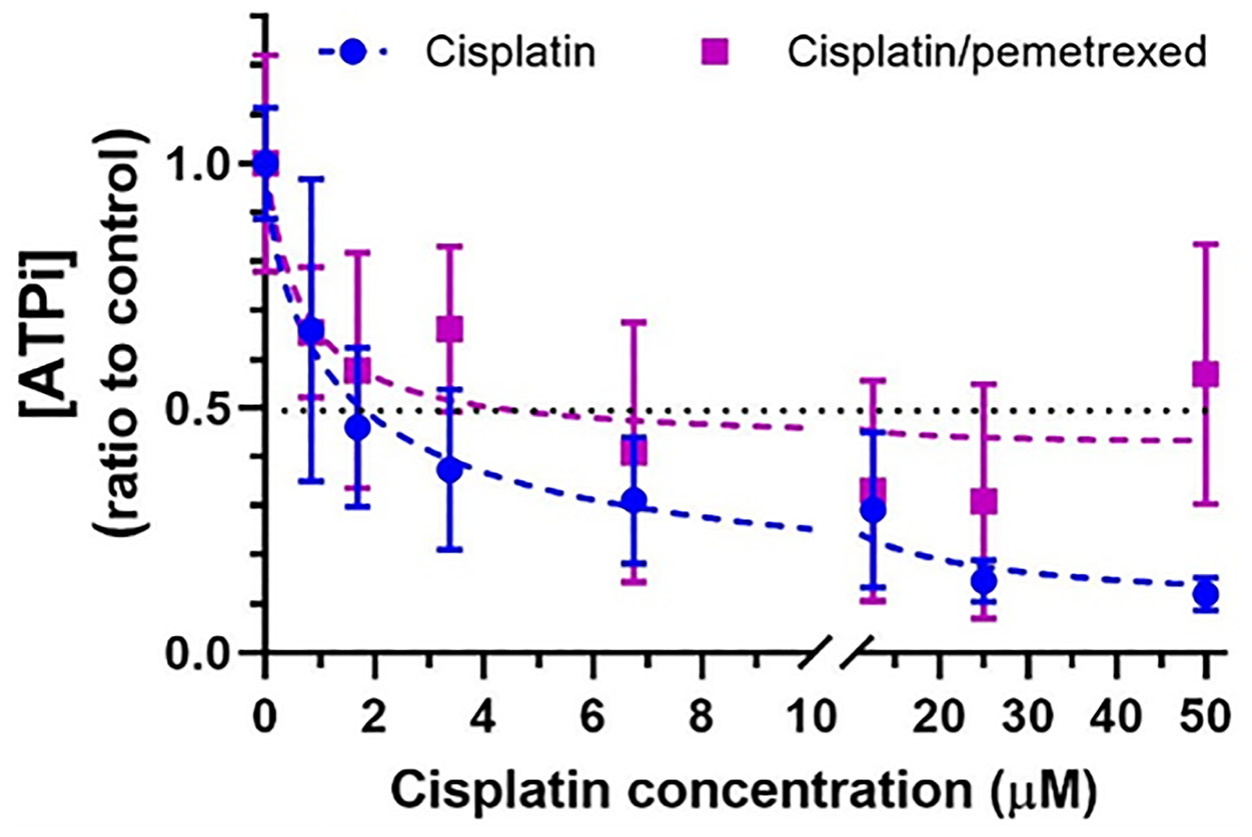
Short-term effect of cisplatin or cisplatin/pemetrexed on viability of H2052/484 spheroids. H2052/484 spheroids (1,000 cells/spheroid) were untreated (control, black curve) or treated with compound for 3 hours at day 3 post-seeding. After washes, the spheroids were cultured for 24 hours and the ATPi levels were determined. Data are presented as the ratio of the ATPi levels of treated spheroid to control spheroid. The data represent the mean ± SD of 2 independent experiments.

The IC50 of both treatments were estimated on H2052/484 spheroids treated for 3 hours with increasing concentrations of cisplatin (from 0 to 100 μM) or the cisplatin/pemetrexed combination (ratio 1:4), and the ATPi levels were measured 24 hours after stopping the treatment (Figure 6).

A 3-hour treatment was sufficient for a dose-dependent decrease of the ATPi levels to be measured 24 hours after the end of the treatment with both cisplatin and the cisplatin/pemetrexed combination. The calculated IC50 was 1.21 μM for cisplatin and 3.18 μM/12.72 μM for the cisplatin/pemetrexed combination.

## DISCUSSION

This study detailed the main steps to obtain tumor spheroids from mesothelioma and lung cancer cell lines, to analyze their growth kinetics and viability, and to assess their chemosensitivity to anti-cancer treatments.

Tumor spheroid cultures have several distinguishing characteristics, including the presence of chemical gradients (oxygen, nutrients, or catabolites) at diameters as small as 200 μm and the development of a central secondary necrotic area at diameters greater than 500 μm.

Cells in the spheroid periphery reflect the *in vivo* situation of actively cycling tumor cells adjacent to capillaries, whereas cells in the center become dormant and eventually die via apoptosis or necrosis.

However, the dependability of the data provided by these models is dependent on their use within a system that is carefully monitored to ensure that bias is kept to a minimum. There are a number of critical issues associated with the use of these models, including the selection of the 3D culture method, the production of homogeneous-sized spheroids, and the identification of the best cytotoxicity test to assess treatment efficacy.

The evolution of cancer treatment toward a precision-based approach has brought significant progress in cancer therapy. Despite promising advancements in molecular driven treatments, disappointing results were obtained in several clinical trials that used targeted therapy, highlighting the limitations of precision medicine. An efficient clinical tool that can predict whether the tumor of an individual patient will be sensitive to a given therapy without developing a resistance is needed.

Here, we have determined the experimental conditions for obtaining 3D spheroids *in vitro* with several established human mesothelioma cell lines and primary cells isolated from patients with lung adenocarcinoma. We have further shown that with this *in vitro* model the anti-cancer effect of both first-line treatment of mesothelioma can be determined, so it represents a useful preclinical model of anti-cancer drug screening.

Our results indicated that spheroid phenotype (loose or tight spheroid, regular or irregular shape), size, and growth differed vastly as a function of two variables: the initiating cell density and the cell type. For almost all the cell populations studied, a phenomenon of compaction was observed at the beginning of the development of the spheroids (from day 1 to day 3 after seeding), as previously described in the literature [33, 34]. It corresponds to an aggregation of cells by the establishment of intercellular bonds involving adhesion molecules. After this step, the size of the spheroid increases due to the proliferation of cells in its outer layer. In the absence of the tumor microenvironment, particularly the vascularization and the fibroblasts, the cells die. The growth curve of spheroids was different for each cell population, with a disaggregation process initiated between day 13 and day 27. This result may reflect variability in the expression of cell receptors (including integrin receptors) and is likely to be cell-type specific. In this study, we determined the minimal number of cells required to assess the growth and the viability of the spheroids. Seeding 1000 cells/spheroid was sufficient to form a spheroid with a diameter of 100 μm at day 3 for all the cell populations studied. This number of cells allowed us to assess the kinetics of the growth of the spheroids, without disaggregation, from day 3 to day 15.

The growth curve of the spheroids in culture and the effect of treatment on this growth were assessed with two parameters: the measurement of the size of the spheroids and the measurement of the ATPi. The size of the spheroids is dependent on the number of cells in the spheroid. The ATPi level of the spheroids is correlated with the number of living cells and with the metabolic activity of the cells. A decrease in this parameter could reflect a decrease in the metabolic activity of the tumor cells incubated with an anti-cancer drug before any changes in the cell number in the spheroid. The two parameters are complementary. A combined measure using the change of spheroid size and the ATPi levels appears to be a robust measure of response for studying chemosensitivity to anti-cancer drugs. Using this method, a concentration-dependent inhibition of H2052/484 spheroid growth and viability was seen with cisplatin and the cisplatin/pemetrexed combination, as previously described with MPM spheroids [30, 35–37].

In conclusion, we established a simple, reliable *in vitro* 3D tumor spheroid model that can be used to characterize the efficacy of anti-cancer drugs on primary cells derived from a patient diagnosed with mesothelioma or lung cancer.

In the future, we plan to adapt our spheroid model to needle biopsy specimens miniaturizing the formation of uniform spheroids across microtiter 384-well plates and improving the quantification of spheroid size and viability. Another step concerns the establishment of co-cultures of tumor spheroids with the cells of the microenvironment (fibroblasts, immune cells). Solid tumors are characterized by a very complex and heterogeneous structural organization in which cancerous cells and stromal cells (i.e., infiltrating immune cells, endothelial cells, cancer-associated fibroblasts) are strongly interlaced and dynamically interact with each other. Direct physical interaction between stromal and cancer cells, as well as the secretion of chemokines, cytokines, and extracellular vesicles, can reconstitute *in vitro* the microenvironment signalling network found *in vivo*, regulating the capability of tumors to grow, metastasize, skip immune regulation, and acquire drug resistance (reviewed in [38]). In the 3D spheroid model, cancer cells can be cultured with other stromal cells such as immune cells [39–41], fibroblasts [42, 43] and endothelial cells [44, 45], to recapitulate the specific tumor tissue heterogeneity found *in vivo*.

In the future, we plan to co-culture tumor spheroids with the autologous immune cells in order to test the efficacy of immunotherapy, the gold-standard treatment for lung cancer.

## MATERIALS AND METHODS

The study was conducted according to the guidelines of the Declaration of Helsinki [46], and approved by the Swiss Ethics Committee on research involving humans (2018–02395, approved in 2018).

### Cells and cell cultures

The human MPM cell lines H2052 (NCI-H2052), and H2452 (NCI-H2452) were purchased from American Type Culture Collection (Manassas, VA, USA). H2052/484 cells were obtained as described previously [47].

The lung adenocarcinoma cells LuCa1, LuCa61, and LuCa62 were established and characterized in our laboratory from human lung tumor resected in the thoracic surgery division of the University Hospitals of Geneva. Surgical samples were obtained from patients with untreated lung adenocarcinoma after informed consent. The biopsy samples were processed for cell culture immediately after surgical resection. Sample from patient 1 (LuCa1; female, 79 years old, NSCLC adenocarcinoma, stage T4 N0 M0 (AJCC, 8^th^ edition)) were cut into small pieces (approximately 1 mm). They were cultured in complete medium composed of Roswell Park Memorial Institute (RPMI) medium supplemented with 10% (v/v) fetal bovine serum, 10 μM HEPES, and antibiotics (complete RPMI, Life Technologies, Carlsbad, CA, USA). After 4 days of culture, some adherent cells were observed in the wells and tissue pieces were removed. After 5 weeks of culture, colonies of epithelial cells were isolated by scraping all other cells in the dish with a rubber policeman, washed with Hank’s Balanced Salt Solution (HBSS; Sigma-Aldrich Chemie GmbH, Buchs, Switzerland) and then detached with trypsin-EDTA (Sigma-Aldrich Chemie GmbH, Buchs, Switzerland). Detached cells were plated and allowed to grow to confluence.

The biopsy samples from patient 2 (LuCa6; male, 72 years old, NSCLC adenocarcinoma, stage T2a N0 M1 (AJCC, 8^th^ edition)) were cut into small pieces and then enzymatically digested in medium supplemented with 0.3 mg/ml collagenase type I, 0.1 mg/ml collagenase type II, 0.025 mg/ml elastase, and 25 mg/ml DNAse for 1 hour at 37°C. The cells were filtered through a 100 μm cell strainer, washed, lysed in red blood cell lysis buffer, and resuspended in complete RPMI medium. After 3 weeks of culture, one epithelial colony was identified, selectively detached with trypsin-EDTA using a cloning cylinder, and cultured. These cells were designated LuCa61. One week later, several epithelial colonies were identified in the same petri dish containing all the digested cells from patient 2. These colonies were isolated, washed with HBSS, and then detached with trypsin-EDTA. They were designated LuCa62.

LuCa1, LuCa61 and LuCa62 cells were maintained in complete RPMI medium up to a passage of 40.

Lung adenocarcinoma cells were characterized according to the expression levels of lung markers in cells cultured in monolayer or spheroids using RT-qPCR. Data were presented in the supplementary materials (Supplementary Figure 2).

All cells were confirmed to be negative for mycoplasma every 2 months, by PCR analysis. For all experiments, LuCa cells were used between passages 3 and 10.

### Spheroids

Between 1000 and 100000 cells suspended in a 200 μL of complete RPMI medium were seeded into each well of a 96-well tissue culture plate (Corning, NY, USA) coated with 50 μL of 1.5% (w/v) agarose (Sigma-Aldrich Chemie GmbH, Buchs, Switzerland). Spheroids were grown at 37°C, at 5% CO_2_. Twice a week, 100 μL per well of medium was replaced with fresh medium.

Spheroids were imaged at the different time points in brightfield with a 4x objective with an inverted microscope (Nikon TS2 FL; Nikon AG, EGG Switzerland). The growth of spheroids was determined by measuring their diameter using a calibrated scale of the microscope and Publisher and Excel softwares (Microsoft, Redmond, WA, USA).

### Metabolic activity assay

Cell viability was measured using the luminescent-based cell metabolic activity assay ATPlite™ (ATPlite™ 1stSTEP, PerkinElmer AG, Schwerzenbach, Switzerland), by the R.E.A.D.S. Unit of the Faculty of Medicine, according to the manufacturer’s instructions modified as follows. Spheroids were transferred to a black 96-well microplate (Corning, NY, USA) with one spheroid per well to a final volume of 80 μL/well. To each well was added 80 μL of the substrate solution, and the spheroids were shaken for 5 minutes in an orbital shaker (Orbi-ShakerMP, Benchmark Scientific, Sayreville, NJ, USA) at 700 rpm. To complete the lysis of spheroids, the plate was sonicated for 5 minutes in an ultrasonic waterbath (Branson ultrasonic SA, Carouge, Switzerland). The intensity of the luminescence signal was detected with a FDSS μCELL plate reader (Hamamatsu Photonic, Solothurn, Switzerland).

### Treatments

Cisplatin (Cisplatine TEVA 100 mg/100 mL; Teva Pharmaceutical Industries, Petah Tikva, Israel) was diluted in sterile NaCl 0.9% at 904 mM. Pemetrexed (Tocris Bioscience, Bristol, UK) was dissolved in sterile DMSO (Sigma-Aldrich Chemie GmbH, Buchs, Switzerland) at concentrations of 10 mg/ml. The cisplatin-pemetrexed combination was made by mixing cisplatin and pemetrexed in a 1:4 ratio (cisplatin/pemetrexed). After 3 days of culture, the H2052/484 spheroids (1000 cells/spheroid) were incubated with cisplatin or cisplatin/pemetrexed combination for 3 hours. Control spheroids were incubated for 3 hours in culture medium alone (control of the cisplatin treatment) or supplemented with 4% DMSO (control of the cisplatin/pemetrexed treatment), the highest concentration of DMSO obtained for the highest concentration of cisplatin/pemetrexed (200 μM/800 μM). After washes, spheroids were cultured for an additional 1 or 17 days. Cell growth and viability were analyzed as previously described. Curves of changes in volumes and intracellular ATP were obtained using Graph Pad Prism 7 software (GraphPad Software, Inc., La Jolla, CA, USA).

### Statistical analysis

Results are presented as mean ± SEM or SD as indicated. The Kruskal-Wallis test was used to examine statistical differences between three or more groups. Statistical differences between pairs of groups were determined using the unpaired Mann-Whitney U test. A *P* value < 0.05 was considered statistically significant.

## SUPPLEMENTARY MATERIALS



## Abbreviations

ATPi: intracellular adenosine triphosphate
2D: two-dimensional
3D: three-dimensional
DMSO: Dimethyl sulfoxide
EDTA: Ethylenediaminetetraacetic acid
HBSS: Hank’s Balanced Salt Solution
HEPES: 4-(2-hydroxyethyl)-1-piperazineethanesulfonic acid
IARC: the International Agency for Research on Cancer
MPM: malignant pleural mesothelioma
