# Supplementary material for: Optimization of tumor spheroid model in mesothelioma and lung cancers and anti-cancer drug testing in H2052/484 spheroids
Source: Oncotarget. 2021 Nov 23;12(24):2375–87. doi: 10.18632/oncotarget.28134 (PMC8629400; doi:10.18632/oncotarget.28134)
Supplement: Supplementary file 1 [file oncotarget-12-2375-s001.pdf]

Optimization of tumor spheroid model in mesothelioma and lung cancers and anti-cancer drug testing in H2052/484 spheroids

SUPPLEMENTARY MATERIALS

The size of H2052/484 spheroids incubated with cisplatin or cisplatin/pemetrexed combination were dose-dependently reduced compared with those of non-treated spheroids. 17 days after the treatment, we observed a huge shrinkage or a complete disintegration of the H2052/484 spheroids (Supplementary Figure 1) for the highest concentrations of cisplatin and cisplatin/pemetrexed combination.

Lung adenocarcinoma cells LuCa1, LuCa61, and LuCa62 were established in our laboratory from human lung tumor resected in the thoracic surgery division of the HUG. They were characterized studying the expression levels of lung markers in cells cultured in monolayer or spheroids using RT-qPCR (Supplementary Figure 2). The A549 lung adenocarcinoma cells were used as control.

The expression of KRT5, KRT7, KRT14, STFC, SOX2, NKX2.1, SCGB1A1, TP63, GAPDH, GUSB, EEFLA1 and TBP mRNAs was evaluated by quantitative RT-PCR as previously described. Primer sequences for the targeted human genes are available upon request.

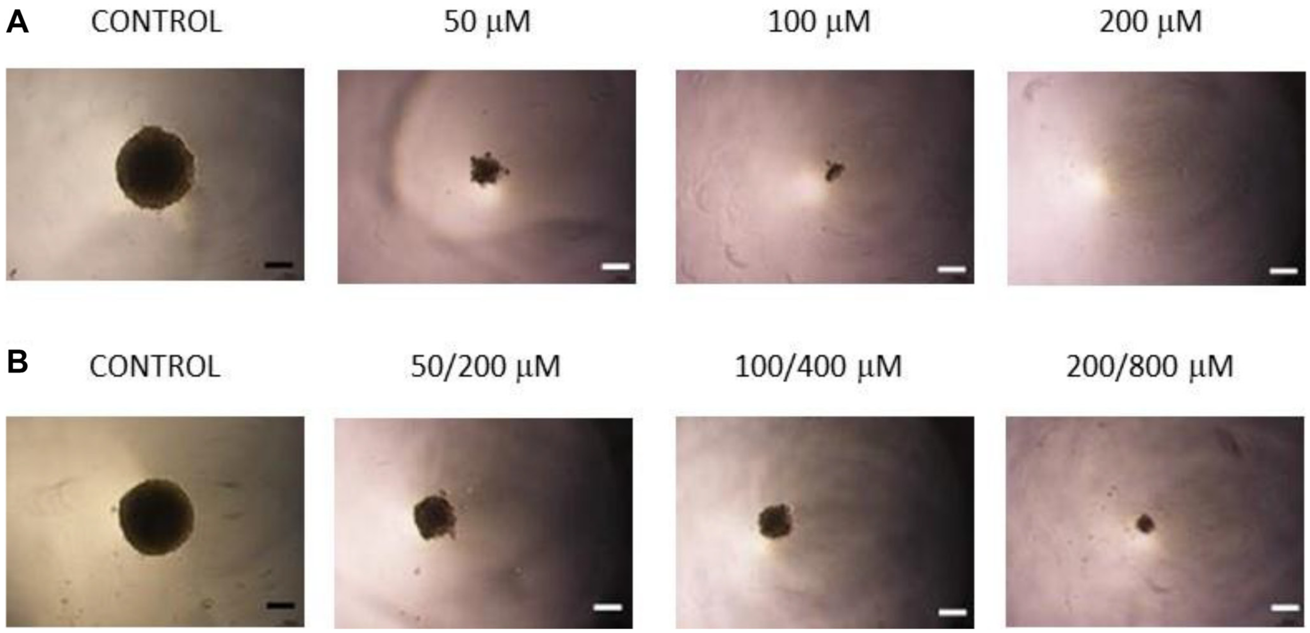

Supplementary Figure 1: Representative images of H2052/484 spheroids 17 days after treatment with (**A**) cisplatin or (**B**) cisplatin/pemetrexed combination. Bar: 100 µm.

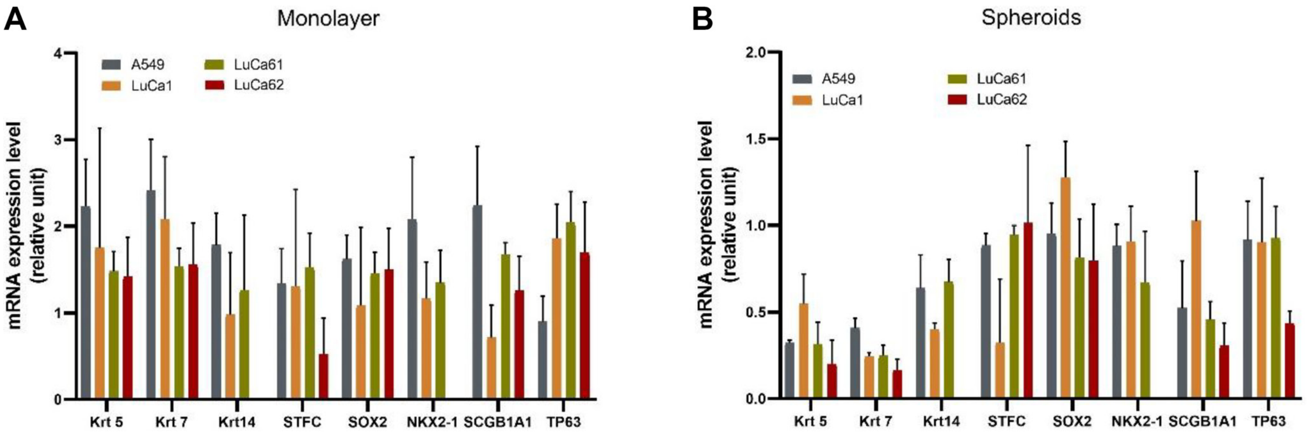

Supplementary Figure 2: Lung adenocarcinoma cell characterization. Lung markers expression was assessed by RT-qPCR in monolayer (**A**) and spheroid (**B**) cultures. A549 cells were used as control. Results were normalized to the expression levels of GAPDH, GUSB, EEFLA1 and TBP expression genes, used as housekeeping genes. The data represent the mean ± SD of 2 replicates.
